# Tinned and Preserved Foods

**Published:** 1906-06-23

**Authors:** 


### TINNED AND PRESERVED FOODS.

### DIFFICULTIES, POISONING, AND A MORAL.

Mr. Upton Sinclair's book has certainly brought to light social and dietetic horrors in Chicago of which few of us could in the most ghastly nightmare even have dreamt. The Packingtown joke that all is used of the pig but the squeal seems literally true, and although the public may relish decomposing game and cheese, they prefer to choose their own variety of decomposition while it is *en train*, and do not care to have it chosen for them and then masked by artificial means and enclosed in tins. Nevertheless all preserved meat does not emanate from Chicago, and we must be careful not to condemn the whole on account of the even masked delinquencies of a part. The exigencies of modern existence render the preservation of meat an economic necessity. Highly populous manufacturing centres must be fed from a distance, and articles of food are perishable, and hence, in the vast majority of cases, without some artificial means is used to keep them, will not stand in transport.

### Preservatives and Colouring Matter in Food.

The economic side of this question is tersely epitomised in one of the paragraphs of the Report of the Departmental Committee upon Preservatives and Colouring Matter in Food which was published in 1901. This paragraph is so germane to the present subject that we think it is worth reproducing *in extenso*: "It should be borne in mind that under the conditions in which the population of Great Britain lives, and more particularly that portion of it inhabiting large towns, some preserving agent, not necessarily chemical, appears to be needed in the case of no inconsiderable portion of its perishable food-supply. It is common knowledge that the food-producing capabilities of this country do not suffice in all particulars for the needs of its population. Under these circumstances the total prohibition of preserving methods would clearly be likely to be attended by serious results to the public health in that large quantities of food possessing highly nutritive value might in effect either be withheld from the poorer classes, or be liable to be consumed by them in a condition of incipient putrefaction."

### No Legal Safeguard.

We know that the Chicago tinned meat revelations will have the effect of prejudicing the sale of all tinned meats and preserved foods and thus hampering, at least temporarily, an industry of extreme economic value. We think this would be to the detriment of the public health and that what is wanted is the control and not abolition of the preserved-meat industry. At the present time the consumer is, except in case of gross decomposition, protected by his own special senses only. Consignments of preserved meat (tins) are examined superficially at the port of entry, but provided the tins do not bulge, showing the absence of the evolution of gas, and do not smell, no further tests are applied, nor, indeed, if one considers the enormous quantity dealt with, can be. As the law of the country stands at the present time, to obtain a conviction under the Sale of Food and Drugs Act, the local authority prosecuting must prove either that the article sold was not of the nature and substance demanded, or that it contained some added ingredient injurious to health. As a matter of fact, either of these is exceedingly difficult to prove, the first implies the existence of a standard, and the second invariably gives rise in the courts to such conflicting evidence that a decision by a police magistrate or quarter sessions is rendered very difficult. The question of a standard is easily to be got over by labelling. If a label is delivered on or with the article sold to the effect that it contains certain added ingredients, or that it is this, that, or the other, it must be held that, or at least can be plausibly argued that, the consumer knows what he is buying and hence cannot be legally, even if he is dietetically prejudiced.

### Toxicological Difficulties.

If we turn from the legal difficulties to the toxicological ones, we find that these latter are not much less complex than the former. In what way, it may be asked, is a consumer likely to suffer from having consumed a tin of Chicago meat, beef, mutton, minced chicken, sausage, or ham prepared under the most filthy and revolting conditions described by Mr. Sinclair? In order to eliminate the æsthetic element, we will suppose that he is in ignorance himself of the method of preparation. The human gastro-intestinal canal is proof against much infection and to-day, knowing what we do of the nature and preparation of much of our food, the trueness of the sacred words "not that which goeth into the mouth defileth a man" is more cogent than ever. The first injury which might accrue to the consumer of inferior preserved meat is malnutrition. This might arise simply from the fact that the tinned meat in question was actually chemically deficient in those elements of nutrition which it should contain. The usual adulterant, for such we may call it, of preserved and, indeed, fresh foods of this nature is water. Water is necessary to the animal body but as a nutrient is useless; it supplies no chemical energy. Now the quantity of water in preserved and fresh foods varies within very wide limits, in sausages for instance, from 60 per cent. to 20 per cent.; in butter from 9 per cent. to 25 per cent. Now, clearly, if a consumer habitually receives and pays the same price for, say, a preserved meat containing 30 per cent. of water as his neighbour does for one containing 20 per cent., the former may clearly regard himself as prejudiced to the extent of 10 per cent. *Ex nihilo nihil fit*, if the chemical nutrient elements are not there, however good the consumer's digestion may be he clearly cannot assimilate them.

### Chemical and Dietetic Values.

We speak advisedly of chemical elements because the analytical chemical data with regard to a given food do not by any means always express its dietetic value. A pair of old boots, a pinch of salt, a drink of water, and a mouthful of atmospheric air contain all the chemical elements of a typical diet, and in ample quantity, yet these dishes would make a poor meal. In meat the principal nutrient element is nitrogen, and hence we class it as a nitrogenous food. But if we are to get the value from this nitrogen it must be present in a form we can digest and subsequently assimilate. Horns, hoofs, cartilage or gristle, skin tendon, and many other parts of an animal's carcase are very rich in nitrogen—richer, if anything, than more tender parts of the flesh—but the human digestion is only partially, if at all, able to extract from the former structures their nutritive elements. From this it follows that the consumer of tinned meat can be, and probably is, prejudiced by the above-mentioned parts of the animal carcase being mixed, flavoured, and sold as meat.

### Potential Sources of Poisoning.

To pass from the question of what may be termed physiological prejudice to the consumer by the ingestion of tinned meats, to pathological prejudice, or to the risks he runs of actual poisoning, we find upon examination that there exist in tinned meat—such as that we believe to have issued from the packing houses in Chicago—three possible potential sources of poisoning. Assuming the cooking of this meat has been insufficient to destroy all pathogenic organisms or their spores, it may contain the active living agencies or active agencies capable of developing the specific bacterial diseases, such as tuberculosis, typhoid, etc. The fact that these products, however unæsthetically they may be prepared, are subjected to high temperatures renders them *a priori* unlikely to be sources of this nature of infection. All preserved meats and sausages contain bacteria, but the above remarks apply to pathogenic or disease-producing bacteria. Deetjen and others examined the bacterial contents of Lyons sausages, and found that 15 grains contained no less than 1,894,000 bacteria; these were reduced, after thirty minutes' boiling, to 9,000—still a goodly number. The two remaining sources of poisoning in preserved meats may be designated chemical. Since the work of Panum, Pouchet, Gautier, and Brieger our attention has been drawn to a series of complicated chemical substances in meat which have been termed ptomaines; some of these are poisonous, and some not. These substances result from the action of different kinds of bacteria on dead flesh, and are not appreciably altered by heat. From this it follows that even the prolonged cooking of flesh which for any reason has become infected with ptomaine-forming bacteria, although it may kill the bacteria and prevent their forming more ptomaines, it will not affect the properties of those already formed. The present article is far too short to enter into the varieties and composition of these substances, but suffice it to say that their identification and isolation is a very difficult matter chemically. The poisonous ones are very active, and hence need only be present in very small quantities to produce disastrous results. A well-known member of this class appears to act like atropine or the poisonous alkaloid derived from the deadly nightshade.

### Poisons Unaffected by Cooking.

The last class of poisons, which may be present in preserved meat, and which will also be quite unaffected by the process of cooking are those chemical substances which are added to it to colour it or to preserve it. This whole question was considered by a Departmental Committee of the Local Government Board, which was presided over by Sir Herbert Maxwell, and which contained Dr. Tunnicliffe as its pharmacologist. This report was issued practically five years ago, but certainly contains all that was known upon this most important economic question up to the time of its publication, and indeed in its appendices embraces original work mostly by Dr. Tunnicliffe concerning the effect upon the human subject of these preservatives. The general tone of the report and the main *motif* of the committee's recommendations is that chemical preservatives should be controlled and not prohibited. The main preservatives used in the tinned meat trade are preparations of boracic acid and a class of bodies called the sulphites, or substances the antiseptic principle of which is $SO_2$. The use of the so-called boron preservatives is very general in all imported preserved and cured meats. For instance, 93 per cent. of all imported hams, 91 per cent. of imported bacons, and 50 per cent. of imported sausages contain these substances. Bisulphite of lime is also largely used in the sausage and preserved meat trade; this substance not only preserves the meat, but also improves to some extent the colour. The use of these substances has increased *pari passu* with the public distaste for salt; preserved meats and ham now contain less salt and saltpetre than formerly. The pickle of which we hear much in Mr. Sinclair's book, and which is undoubtedly, and we venture to think in the majority of cases legitimately, injected into carcases consists essentially of 5 lb. of boracic acid, 55 lb. of salt, 5 lb. of saltpetre, and 5 lb. of cane sugar, dissolved in 20 gallons of water.

### Smoking as a Preservative.

We cannot leave this subject without drawing attention to the preservative action of smoking, probably due to pyrrol, pyridene and allied substances having a very high antiseptic power. Some of the products of smoke are highly complicated, and we have deep sympathy with an analytical chemist who is called upon to analyse a kipper. The question of colouring matter does not seem very important from the point of view of the public health. Although it is quite certain that aniline dyes are used to colour hams, sausages, skins and substance, and other preserved meats, the enormous colouring power of these substances renders their use necessary in only the most minute quantity, and that any harm will accrue from these very small quantities is very unlikely.

### Dangers and a Moral.

The facts discussed above show clearly to the merest tyro how complicated this question is from the scientific standpoint; still more complicated is it when we consider that the preservation of food has also a political aspect. There are undoubtedly those who, to protect certain vested interests, would wish to prohibit all those processes which save waste, and amongst them the preservation and open import of preserved food. The effect of this would simply be to raise the price of raw food material to the masses —a most undesirable result.

The moral we must draw is simply that though the Chicago packing houses have shown themselves disgusting, and perhaps even poisonous, there are many houses both in our colonies and at home that turn out preserved meats of the most excellent quality at a most reasonable price, and not only do a very good trade by adopting honest methods, but also perform a most valuable economic function.

The second lesson that we may learn from a consideration of the above details is how difficult it is to absolutely protect the consumer by examination of the finished product, and how much easier it would be to do so by proper inspection of materials at the source of the supply.

